# Prevalence, Risk Factors and Social Context of Active Pulmonary Tuberculosis among Prison Inmates in Tajikistan

**DOI:** 10.1371/journal.pone.0086046

**Published:** 2014-01-20

**Authors:** Daniel E. Winetsky, Olga Almukhamedov, Dilshod Pulatov, Natalia Vezhnina, Aizhan Dooronbekova, Baurzhan Zhussupov

**Affiliations:** 1 Department of Medicine, University of Pennsylvania, Philadelphia, Pennsylvania, United States of America; 2 Department of Programs, AIDS Foundation East-West, Dushanbe, Republic of Tajikistan; 3 Department of Programs, AIDS Foundation East-West, Almaty, Republic of Kazakhstan; 4 Department of Monitoring and Evaluation, AIDS Foundation East-West, Almaty, Republic of Kazakhstan; St. Petersburg Pasteur Institute, Russian Federation

## Abstract

**Setting:**

Tuberculosis (TB) is highly prevalent in prisons of the former Soviet Union.

**Objective:**

To understand the behavioral, demographic and biological factors placing inmates in Tajikistan at risk for active TB.

**Design:**

We administered a behavioral and demographic survey to 1317 inmates in two prison facilities in Sughd province, Tajikistan along with radiographic screening for pulmonary TB. Suspected cases were confirmed bacteriologically. Inmates undergoing TB treatment were also surveyed. In-depth interviews were conducted with former prisoners to elicit relevant social and behavioral characteristics.

**Results:**

We identified 59 cases of active pulmonary TB (prevalence 4.5%). Factors independently associated with increased prevalence of active TB were: HIV-infection by self-report (PR 7.88; 95%CI 3.40–18.28), history of previous TB (PR 10.21; 95%CI 6.27–16.63) and infrequent supplemental nutrition beyond scheduled meals (PR 3.00; 95%CI 1.67–5.62). Access to supplemental nutrition was associated with frequency of visits from friends and family and ability to rely on other inmates for help.

**Conclusion:**

In prison facilities of Tajikistan, HIV-infection, injection drug use and low access to supplemental nutrition were associated with prevalent cases of active pulmonary TB. Policies that reduce HIV transmission among injection drug users and improve the nutritional status of socially isolated inmates may alleviate the TB burden in Tajikistan’s prisons.

## Introduction

**Figure 1:**
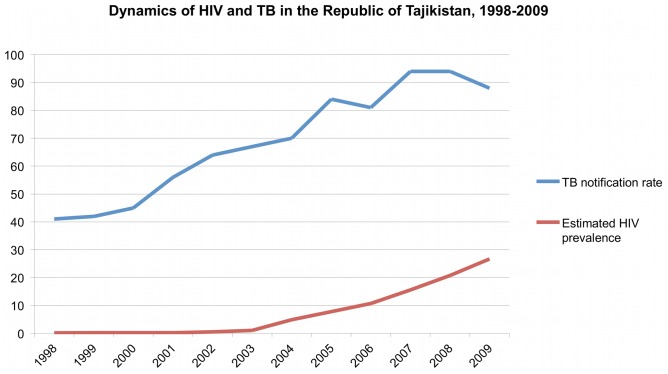
Prevalence of HIV (registered cases per 100,000 population) and WHO notification rates for TB (cases per 100,000 person-years) in the republic of Tajikistan from 1998–2008 [1] [41].

Since the early 1990s, the incidence of active tuberculosis in the formerly Soviet Central Asian Republics has risen dramatically [1]. In the Republic of Tajikistan, where per capita GDP is among the lowest of any former Soviet republic [2], estimated TB incidence has more than doubled since 2000 (Figure 1). According to drug resistance surveys, rates of multidrug-resistance in Tajikistan are among the highest in the world [3]. Furthermore, the recent rise in HIV prevalence threatens to further exacerbate this epidemic (Figure 1).

Prisons may have played a role in the epidemiology of tuberculosis in this region [4]. Places of incarceration present unique challenges to the control of tuberculosis, because of overcrowding and prolonged exposures. Because risk behaviors for HIV transmission are common among those subjected to incarceration, inmates may be particularly susceptible to HIV/TB co-infection [5]. Furthermore, the high mobility of inmates between the general population and the prison population makes correctional facilities an important social vector for disease transmission. In Eastern Europe and Central Asia, increased rates of incarceration are associated with civilian rates of multidrug-resistant TB and have been implicated in increased TB incidence in the general population [4].

Individuals in Tajikistan’s penal institutions are subject to interacting social and economic forces that contribute to their vulnerability to infectious diseases. The Republic of Tajikistan has an estimated population of 7.0 million and a per capita GDP of $1,791 [2]. The fall of the Soviet Union in 1991 and a subsequent civil war from 1992 to 1997 left Tajikistan’s economic and public health infrastructure in disarray [6]. Since that time, patterns of labor migration (mainly to Russia and Uzbekistan) and drug transit through the region have contributed to rising prevalence of injection drug use and HIV-infection in Tajikistan’s population [7], [8]. The region has also been subject to the development of resistant strains of tuberculosis, likely resulting from health sector dysfunction. In a 2009 drug resistance survey performed in Tajikistan, an estimated 16.5% of new cases and 61.6% of retreatment cases exhibited multidrug-resistance [3]. Resistance surveys among incarcerated individuals have not been conducted in Tajikistan, but prevalence of multidrug-resistance of up to 27–38% of new cases has been reported in prisons elsewhere in the former Soviet region [9]–[11].

In studies of active TB among incarcerated populations of the former Soviet Union from the last decade, prevalence has ranged from 1.2% to 9.9% [12]–[16]. Active TB disease in these settings has been associated with a prior history of TB treatment, illicit drug use, overcrowding and poor access to clean sheets [12], [17]. HIV-infection has not been associated with TB in these studies, though prevalence of HIV has risen sharply since many of them were conducted. In the Republic of Tajikistan, the Department of Corrections conducts annual radiographic screening for active pulmonary tuberculosis with the use of mass miniature radiography (MMR). MMR data from 2007 showed 372 new cases of active pulmonary TB were detected among prisoners; however, the exact number of inmates screened is not publicly available. The exact burden of active TB and its associations in the prison system of Tajikistan have otherwise not been studied.

Tuberculosis is widely held to be a disease of poverty. Through social isolation and poor nutritional status, forms of deprivation have been linked to increased incidence of tuberculosis [18] and worse treatment outcomes [19]–[21]. A growing body of literature suggests that interventions targeted to alleviating poverty by providing social and economic support may improve TB outcomes among vulnerable populations [21]–[23]. However, the ways that socio-economic status in the general population may translate to the controlled environment of a prison have not been well characterized.

In this study, we conducted a cross-sectional prevalence survey of active pulmonary tuberculosis among prison inmates in the Republic of Tajikistan, including administration of a behavioral-demographic survey to examine the risk factors and social context for active TB disease.

## Study Population, Materials and Methods

### Study Setting and Population

We conducted a cross-sectional survey in two prison facilities in Sughd province, in the northern part of the Republic Tajikistan. All participants were male and over the age of 18. We conducted the study in January and February of 2010. Both facilities were medium-security barrack-style facilities housing exclusively individuals serving sentences after conviction. According to a national report released prior to our data collection, the physical infrastructure of existing prison facilities at the time of this study dated from the 1940s–1960s, and heating, ventilation and cooling systems were lacking [24].

### Sampling Method

At the time of this study, there were approximately 12,000 prison inmates in a total of 12 facilities in the Republic of Tajikistan housing males. The study was conducted in two facilities with a combined population of approximately 1,350 inmates. These all-male facilities were chosen based on a pre-existing relationship with AIDS Foundation East-West, the principle non-governmental organization responsible for the study. All inmates in these two facilities were invited to participate in our study, including inmates undergoing treatment for active tuberculosis.

### Formative Interviews

In preparation for this survey, we conducted a pilot study involving semi-structured in-depth interviews with former inmates to assess their knowledge, attitudes and practices regarding HIV and TB. We interviewed 14 former inmates, recruited from the members and clientele of a mutual assistance organization in Khudjand, Tajikistan (Sughd province), who had been released from prisons in Tajikistan within the prior 12 months. Interview question guides were developed in accordance with existing guidelines for qualitative studies of HIV and TB [25]–[27]. A content analysis was performed to elucidate common themes among interview responses. A number of themes emerged unexpectedly from these interviews and were explored further in the behavioral-demographic questionnaire developed for our survey.

### Behavioral-demographic Questionnaire

On the basis of themes elicited during semi-structured in-depth interviews, as well as a review of the relevant scientific and theoretical literature [7], [8], [27]–[29], we developed a structured behavioral-demographic questionnaire. The questions on this questionnaire included basic demographic data (age, sex, nationality, education level, etc.), medical history (HIV status, previous history of tuberculosis, lung disease, head and neck cancer, etc.), health and risk behaviors (tobacco, alcohol and drug use, health provider utilization, use of alternative remedies, etc.) and structural/environmental factors (number of cellmates, ventilation of living space, frequency of sheet changes, etc.). Additional questionnaire items were formulated to explore themes arising from in-depth interviews and review of the literature including personal history of labor migration, access to food outside of scheduled meals, and integration with inmate and civilian social networks. Questionnaire items assessing social network integration were designed to evaluate both structural and functional aspects of social support, as both have been linked with health [28], [30], [31]. A similar approach has been used elsewhere to assess inmate social network integration [32]. Questionnaires were administered in person either in Russian, Tajiki or Uzbek, depending on the participant’s preferred language.

### Tuberculosis Case Finding and Diagnostic Evaluation

In combination with administration of the behavioral-demographic questionnaires, participants were screened for current symptoms of active pulmonary tuberculosis, and their height and weight were measured. We coordinated our study with annual MMR screening required by law for all inmates in the Republic of Tajikistan. Two independent physicians reviewed all chest radiographs. On the basis of symptom scores, BMI and chest radiographic findings, sputum samples were collected from participants with suspicion for tuberculosis for bacteriological examination with sputum smear microscopy (using Ziehl Neelsen staining, performed locally in Sughd province) and culture (using quality assured BACTEC MGIT and Löwenstein-Jensen agar, performed at the National Reference Laboratory for Tuberculosis in Dushanbe). A modified version of the World Health Organization (WHO) symptom scoring system was used to select cases for further evaluation [33]. In addition, a subset of 184 inmates had participated in an HIV seroprevalence survey conducted by the Republican Center for AIDS Prophylaxis four months prior to our study. Sputum samples were collected for smear microscopy from these individuals regardless of symptom score or radiographic findings.

All TB suspects were evaluated by physicians from the prison health administration, and all identified cases were treated with a standard DOTS course, in accordance with existing policy of the Ministry of Health of the Republic of Tajikistan. For the purpose of this study our case definition for active smear-positive pulmonary tuberculosis included all individuals found to have two positive sputum smears. Our case definition of active smear-negative pulmonary tuberculosis included all individuals found to have culture-positive, but smear-negative sputum for *Mycobacterium tuberculosis* and all individuals with a clinical diagnosis of active pulmonary tuberculosis, on the basis of symptoms, chest radiographic findings and failure to respond to broad-spectrum antibiotics, consistent with WHO guidelines [33]. Cases of extra-pulmonary TB were excluded from our study.

### Data Analysis

Data from questionnaires and from screening and diagnostic tests were entered into a secured database with the use of Access 2007 (Microsoft, Seattle, WA, USA) and exported to R 2.13.2 (R Development Core Team, 2011) for analysis [34]. For inference regarding individual risk factors, prevalence ratios were estimated using linear regression models with the negative binomial distribution. Mixed effects models were used to account for the clustering of our respondents in two facilities. Multiple imputation was used to handle missing data. Adjusted prevalence ratios were estimated with the use of multivariate regression. A multiple regression model was constructed using a step-wise forward selection approach. Only variables with a statistical significance level of p<0.20 in univariate analysis were considered for inclusion in the multivariate model. Data were analyzed both with and without the inclusion of individuals undergoing treatment at the time of the study or with a previous history of TB.

Unless otherwise stated, analyses were designed to evaluate associations with clinical cases of active pulmonary TB, since only a small proportion of cases were culture-positive. Incidence rate ratios (IRR) were used in all regression analyses where frequency of supplemental nutrition was the response variable. Poisson regression was selected for these analyses, because the distribution of events was highly skewed toward the lower end of the variable range. Difference in ordinal response variables between two groups (e.g HIV-positive vs. HIV-negative) tested with Wilcoxon rank-sum test.

### Ethical Considerations

Informed consent was obtained from all participants. During the informed consent process, interviewers emphasized to individuals that their participation in the behavioral-demographic questionnaire and the use of their clinical data for research was voluntary and separate from the mandatory mass miniature radiographic screening with which our study was coordinated. Though enhanced screening was one of the potential benefits to participation in our study, declining to participate in no way impacted an individual’s access to further diagnostic evaluation and treatment if evidence of disease was detected. Health care workers and NGO employees from the community were recruited to conduct interviews, and rates of participation were similar between community interviewers and interviewers recruited from among prison health care staff. Due to informal social norms amongst inmates that forbid signing documents (and which may at times be enforced violently by fellow inmates), verbal consent was obtained from participants. After explaining the risks and benefits of participating in the study, interviewers asked inmates if they wished to participate and documented each inmate’s response. Copies of the informed consent document were made available to participants. Approximately 400 individuals had previously participated in an HIV seroprevalence survey in the previous year, conducted by the State AIDS Service, and these participants separately consented to the use of their HIV data from that survey; however, this data was ultimately not used for this study due to concerns of inadequate linkage between the two data sets. The verbal consent procedure was explicitly discussed and approved during the ethical board approval process. This research was approved by Stanford University’s Institutional Review Board for human subjects research and by the Ministry of Health of the Republic of Tajikistan.

## Results

### Population Demographics

Of 1,350 inmates invited to participate, 33 declined. All 1,317 inmates surveyed were male. Participants ranged in age from 19 to 71, with a median age of 36, and interquartile range from 29–43. The majority of participants (n = 927; 71.5%) identified their nationality as Tajik (Table 1) and had completed secondary school (n = 939; 71.3%). The current sentence was the first episode of incarceration for the majority of participants (n = 914; 70.8%), and the median length of the current incarceration was 35 months. Demographic characteristics did not differ significantly between the two prison facilities we studied.

**Table 1:** Demographic characteristics of inmates and risk factors for active pulmonary TB.

	N (%)	TB+ n (%)	TB- n(%)	PR (95%)	aPR (95%)
Age group					
18–29	389 (29.6)	13 (3.3)	376 (96.7)	1.0	1.0
30–39	485 (37.0)	25 (5.2)	460 (94.8)	1.54 (0.80–2.97)	1.13 (0.55–2.39)
40–49	316 (24.1)	11 (3.5)	305 (96.5)	1.04 (0.47–2.29)	0.81 (0.33–1.97)
50+	122 (9.3)	10 (8.2)	112 (91.8)	2.45 (1.10–5.45)*	1.52 (0.62–3.73)
Nationality					
Tajik	925 (71.5)	44 (4.8)	881 (95.2)	1.0	1.0
Other	369 (28.5)	15 (4.1)	354 (95.9)	0.85 (0.48–1.52)	0.84 (0.45–1.57)
Highest level of education completed					
Primary school and less	176 (13.6)	6 (3.4)	170 (96.6)	1.0	1.0
Secondary school	938 (72.7)	46 (4.9)	892 (95.1)	1.44 (0.62–3.32)	1.46 (0.58–3.70)
Some college and more	176 (13.6)	7 (4.0)	169 (96)	1.17 (0.400–3.40)	1.54 (0.46–5.14)
Locality prior to incarceration					
Urban	838 (65.2)	38 (4.5)	800 (95.5)	1.0 (0.60–1.72)	0.99 (0.55–1.77)
Rural	447 (34.8)	20 (4.5)	427 (95.5)	1.0	1.0
Family status					
Single	457 (35.4)	20 (4.4)	437 (95.6)	0.94 (0.55–1.59)	0.59 (0.32–1.10)
Married	834 (64.6)	39 (4.7)	795 (95.3)	1.0	1.0
Number of previous sentences					
None	913 (70.9)	36 (3.9)	877 (96.1)	1.0	1.0
One prior sentence	241 (18.7)	16 (6.6)	225 (93.4)	1.68 (0.95–2.98)	1.37 (0.70–2.68)
Two or more prior sentences	134 (10.4)	6 (4.5)	128 (95.5)	1.14 (0.49–2.64)	0.95 (0.36–2.50)
BMI					
<18.5	39 (3.0)	9 (23.1)	30 (76.9)	5.79 (3.07–10.91)*	2.22 (0.98–5.01)
18.5+	1254 (97.0)	50 (4.0)	1204 (96.0)	1.0	1.0
History of international labor migration					
Yes	567 (43.2)	24 (4.2)	543 (95.8)	0.90 (0.54–1.50)	0.98 (0.56–1.72)
No	746 (56.8)	35 (4.7)	711 (95.3)	1.0	1.0
Alcohol consumption prior to incarceration					
At least two times a week	100 (7.8)	2 (2.0)	98 (98.0)	0.45 (0.11–1.81)	0.55 (0.13–2.36)
Weekly	131 (10.3)	9 (6.9)	122 (93.1)	1.53 (0.77–3.05)	0.70 (0.30–1.65)
Two times a month and rarely or none	1046 (81.9)	47 (4.5)	999 (95.5)	1.0	1.0
Tobacco smoker at time of study					
Yes	507 (38.6)	26 (5.1)	481 (94.9)	1.25 (0.76–2.07)	1.43 (0.81–2.54)
No	806 (61.4)	33 (4.1)	773 (95.9)	1.0	1.0
History of drug use through injections					
Yes	55 (4.2)	6 (10.9)	49 (89.1)	2.59 (1.16–5.76)*	1.40 (0.39–5.07)
No	1258 (95.8)	53 (4.2)	1205 (95.8)	1.0	1.0
Frequency of supplemental nutrition					
<1x/month	672 (53.1)	45 (6.7)	627 (93.3)	3.0 (1.67–5.62)*	2.08 (1.06–4.05)*
≥1x/month	594 (46.9)	13 (2.2)	581 (97.8)	1.0	1.0
HIV-status by self-report					
Positive	12 (0.9)	4 (33.3)	8 (66.7)	7.88 (3.40–18.28)*	13.44 (3.77–47.95)*
Negative	1301 (99.1)	55 (4.2)	1246 (95.8)	1.0	1.0
Number of cellmates sharing domicile					
≤69 cellmates	326 (26.0)	20 (6.1)	306 (93.9)	1.51 (0.79–2.91)	1.21 (0.59–2.49)
70–149	556 (44.4)	21 (3.8)	535 (96.2)	0.93 (0.49–1.78)	0.89 (0.44–1.79)
150+	370 (29.6)	15 (4.1)	355 (95.9)	1.0	1.0
History of TB					
Yes	90 (7.0)	24 (26.7)	66 (73.3)	10.21 (6.27–16.63)*	8.22 (4.38–15.44)*
No	1187 (93.0)	31 (2.6)	1156 (97.4)	1.0	1.0

Demographic characteristics of inmates and risk factors for active TB in univariate and multivariate analysis. Prevalence ratios (PR) and 95% confidence intervals (95%CI) were weighted using random effects modeling. Adjusted prevalence ratios (aPR) and their respective 95% confidence intervals (95%CI) were derived from multivariate regression analysis. * p<0.05.

### Active TB Prevalence

We surveyed 1,317 inmates in two prison facilities and identified 59 cases of active pulmonary tuberculosis (point prevalence: 4.5%; 95%CI 3.4–5.7) diagnosed on the basis of clinical and radiographic criteria. Of these, 36 (61.0%) were smear-positive. Sputum culture was positive in 16 cases (27.1%). Forty-seven cases (79.7%) had radiographic findings suggestive of TB. Symptoms of TB were present in 30 (50.8%) cases (Table 2). Disease prevalence did not differ significantly between the two prison facilities we studied.

**Table 2:** Clinical and microbiological characteristics of prevalent TB cases.

	Total	Culture-positive	Culture-negative	Radiographic findings present	Symptoms present
Smear-positive	37 (100%)	5 (14%)	32 (86%)	36 (97%)	17 (46%)
Smear-negative	22 (100%)	11 (50%)	11 (50%)	11 (50%)	13 (59%)

Radiographic findings were defined as any features determined to be suspicious for TB after reading of MMR films by two independent radiologists. In this table, presence of symptoms refers to report by respondents of having any of the following symptoms at the time of the survey: “cough,” “sputum with or without blood,” “weight loss in the last three months,” “loss of appetite,” “chest pain,” “night sweats,” “generalized weakness or fatigue,” “shortness of breath.”

### Factors Associated with Prevalent TB

We explored environmental, biological and behavioral risk factors associated with clinically and radiographically diagnosed prevalent cases of active TB with the use of univariate and multivariate mixed effects negative binomial regression models. Factors associated only with culture-positive cases could not be identified, owing to their small numbers. Prison housing in Tajikistan is mainly composed of barracks, and nearly all respondents reported sharing living spaces with multiple cellmates. The median number of inmates sharing a domicile was 110, with an interquartile range of 69–151. More than one quarter of participants believed they lived with someone who had active TB in the three preceding months (n = 349; 26.5%), but there was only a non-statistically significant trend toward increased prevalence of active TB diagnosis with a presumed TB contact (p = 0.086). Nearly all respondents reported daily ventilation of their living space (n = 1,238; 94.0%). Access to supplemental nutrition less than once per month was associated with a higher prevalence of active TB (PR 3.00; 95%CI 1.67–5.62) (Table 1). Access to supplemental nutrition was defined as the number of times participants reported eating food purchased in the commissary or delivered by friends and family over and above the meals supplied by the prison administration in the 3 months prior to our study, and was assessed as an ordinal variable in our survey.

Previous history of tuberculosis by self-report was strongly associated with a diagnosis of active TB in our sample (PR 10.21; 95% CI 6.27–16.63). HIV-infection by self-report was also associated with an increased prevalence of active TB (PR 7.88; 95%CI 3.40–18.28) (Table 1). Low BMI (<18.5) and advanced age (>50) were also associated with increased prevalence of TB (PR 5.79; 95%CI 3.07–10.91 and PR 2.45; 95%CI 1.10–5.45, respectively) (Table 1). Previous history of diabetes, asthma, lung disease and head and neck cancer were not associated with active TB.

Of 1,317 respondents, 161 individuals (12.2%) reported having used drugs. Of these, 55 individuals (34.2% of drug users) had injected drugs (Table 1). History of injection drug use was strongly associated with having active TB (PR 2.59; 95%CI 1.16–5.76) and with having HIV-infection (PR 16.34; 95%CI 5.36–49.84). Smoking history was prevalent (n = 508; 38.6%), but was not associated with active TB. A substantial number of respondents reported a personal history of labor migration (n = 567; 43.1%), but it was not associated with HIV-infection or active TB disease (Table 1).

In multivariate negative binomial regression, factors independently associated with prevalent TB cases were HIV by self-report (aPR 13.44; 95%CI 3.77–47.95), previous history of active TB (aPR 8.22; 95%CI 4.38–15.44) and low access to supplemental nutrition (aPR 2.08; 95%CI 1.06–4.05). When adjusted for HIV-infection by self-report, injection drug use was not independently associated with increased prevalence of TB. Similarly, the association of low BMI with active disease was not significant in multivariate analysis (Table 1). Risk factors were not substantially different when retreatment cases and individuals already undergoing treatment at the time of screening were excluded, with the exception that the associations of prevalence of TB with low BMI and advanced age were not significant in either univariate or multivariate analysis.

### Perceptions of TB and Health-seeking Behavior

The perception that inmates are at high risk of developing tuberculosis was a common theme among in-depth interviews with former inmates. In our behavioral-demographic survey, 70.3% of respondents (n = 1,264) reported perceiving at least some personal risk of developing tuberculosis (including low, moderate and high risk), and 19.6% perceived themselves to be at high risk. Avoidance of individuals infected with TB was the most commonly cited method for prevention of TB (86.2%; n = 1,317), though 27.2% of respondents reported having shared a living space with someone with active TB (n = 1,282) in the three months prior to our study. Of note, in prison facilities of Tajikistan, individuals with smear-negative TB continue remain housed in barracks with the general population, so this perception may accurately reflect contact history.

The use of alternative remedies was an additional theme arising from in-depth interviews and in our behavioral-demographic survey, 47.0% of inmates reported believing alternative remedies to be somewhat or very effective (n = 1,289). However, a substantial majority of inmates (95.2%; n = 1,317) reported they would seek help from medical personnel if concerned they may be developing tuberculosis. The majority of inmates also reported they had undergone testing for HIV in the past (58.9%; n = 1162).

### Social Context

During in-depth interviews with former inmates conducted in the formative phase of our study, common themes were the importance of maintaining connections to friends and family and of participation in inmate social networks via the trade of goods and services as a means for maintaining health and overcoming deprivation. Examples of statements typifying these themes include:

“I supported my health through relatives. My mother is alive, my little brother, they were alive… they helped, yes… with medicines and with food, there’s no other way you can help in prison.”

“…I’m a craftsman… I earn money and then I get treatment.”

Therefore, in our behavioral-demographic questionnaire, we sought to measure the degree of social integration maintained by participants both with social networks outside the prison facility and with other inmates (Table 3). We then explored the role of these social networks in the matrix of behavioral and biological risk factors for active TB.

**Table 3:** Social characteristics of inmates surveyed and prevalence of risk behaviors among respondents.

Table 3. Social characteristics of respondents				
	Total responding	n (%)	Range	Median (IQR)
Number of visits by friends and family in 6 month period prior to study	1,092		0–100	3 (2–6)
Number of packages received from friends and family in 6 month periodprior to study	1,078		1–100	4 (3–6)
Number of other inmates a respondent could rely on for help if necessary				
0	1,143	82 (7.2)		
1–3	1,143	564 (49.3)		
4–10	1,143	326 (28.5)		
11–20	1,143	101 (8.8)		
21–100	1,143	41 (3.6)		
>100	1,143	29 (2.5)		
Frequency of supplemental nutrition in 3 month period prior to study				
0	1,276	197 (15.5)		
1–3	1,276	476 (37.6)		
4–12	1,276	299 (23.6)		
13–24	1,276	117 (9.2)		
25–72	1,276	58 (4.5)		
>72	1,276	120 (9.5)		

IQR = interquartile range.

**Figure 2:**
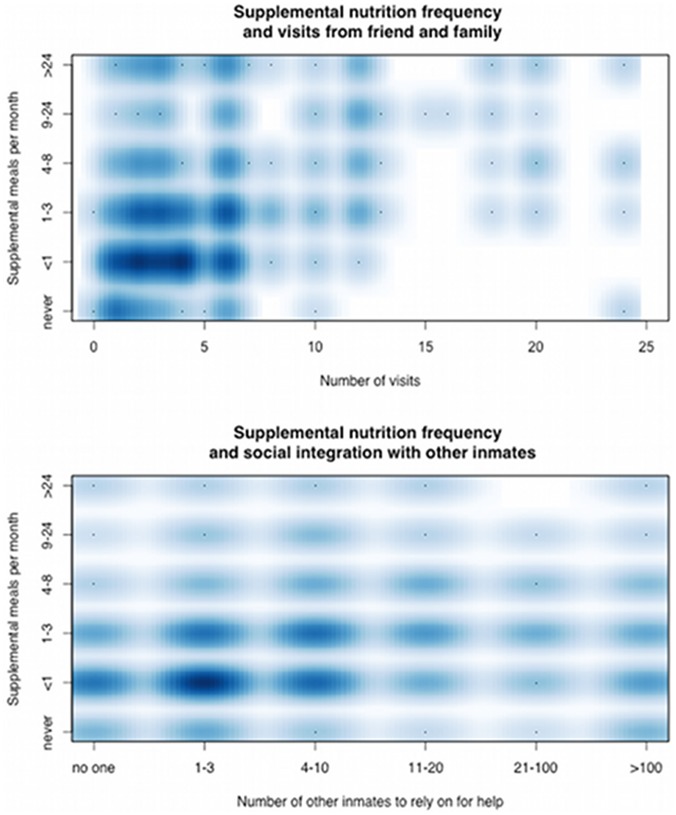
Social context and nutrition. Frequency of supplemental nutrition is correlated with both A) number of visits from friends and family, and B) number of other inmates respondents felt they could rely on for help if necessary, representing social networks outside of and within the prison facilities.

To assess associations of social network variables with supplemental nutrition, incidence rate ratios (IRR) were calculated, indicating the relative incidence of an incremental increase in the frequency of supplemental nutrition over and above meals allotted by the prison administration by one ordinal grouping (Table 3) associated with an additional family/friend visit, an additional delivery, or an incremental increase in social integration with other inmates. In univariate Poisson regression analysis, frequency of supplemental nutrition was associated with frequency of visits by friends and family (IRR = 1.04, p<0.001), frequency of deliveries from friends and family (IRR = 1.04, p<0.001) and with the number of other inmates that individual respondents reported they could rely on for help (IRR = 1.21, p<0.001; see Table 3 for ordinal groupings) (Figure 2).

Visits and deliveries from friends and family were also associated with increased BMI (OR = 1.15, p<0.001 and OR = 0.12, p<0.001, respectively, where odds ratio (OR) indicates the relative odds of an incremental increase of 1 unit in BMI associated with an increase in ordinal level of frequency; see Table 3) in univariate linear regression. However, increased ability to rely on other inmates showed only a trend toward increased BMI that was not statistically significant (OR = 1.44, p = 0.12).

Individuals with HIV by self-report had significantly lower frequency of supplemental nutrition (OR = 0.33, p<0.05, by Wilcoxon rank-sum test), and there was a non-significant trend toward lower frequency of supplemental nutrition among injection drug users (OR = 0.75, p = 0.056), but neither those with HIV nor injection drug users reported less frequent visits or fewer inmates on whom they could rely for help. In linear regression, duration of the current episode of incarceration was negatively associated with a decreased frequency of visits from friends and family (OR = 0.77, p<0.001), but was positively associated with an increased ability to rely on other inmates (OR = 1.34, p<0.05). The majority of respondents reported that an inmate’s relationship to other inmates would be negatively impacted if diagnosed with TB (63.5%; n = 1,286). However, a diagnosis of active TB in our sample was not negatively associated with ability to rely on other inmates for help. Multiple regression analysis to determine independent predictors of supplemental nutrition was not performed due to concerns for multicollinearity.

## Discussion

We estimated a point prevalence of 4.5% for active pulmonary TB on the basis of clinical and radiographic criteria among male inmates in the prison system of Tajikistan. This is comparable to values reported for other prisons within the region [35], [13]–[16] and elsewhere [36]. Such a high burden of active TB, much of which is likely to be multidrug-resistant, represents a substantial threat to public health, and may be an enormous financial challenge for the already strained health infrastructure of the former Soviet republic with the lowest per capita GDP. Since the completion of data collection for this study, substantial efforts have been made to enhance infection control in the penitentiary system of Tajikistan. Sustaining such efforts in Tajikistan, and other high burden countries with constrained national budgets, will depend on the continued financial and technical support of the international community.

Factors independently associated with an increased prevalence of active TB in our sample were a previous history of TB, self-reported HIV-infection and poor access to supplemental nutrition beyond the meals supplied by the prison administration. Injection drug use, low BMI, advanced age and low number of cellmates were also significantly associated with active TB in univariate analysis, but were not independent predictors in multivariate regression. Low number of cellmates also ceased to be predictive when individuals already enrolled in TB treatment were excluded from analysis, suggesting this association was incidental and related to their isolation in the infirmary. The associations of prevalent cases of TB with previous TB history and injection drug use are consistent with findings from other former Soviet prison facilities [12], [37]. HIV-infection has not previously been associated with TB cases in prevalence studies of prison facilities from the region but high rates of HIV have been reported among inmates with TB compared with civilian cases of TB [17], and has been associated with multi-drug resistance among incarcerated individuals [38]. Our finding that low access to supplemental nutrition was associated with TB prevalence has not been previously reported among inmates and warrants further investigation.

*Social context and supplemental nutrition*:

To understand access to supplemental nutrition in Tajikistan’s prison facilities requires an understanding of potential sources of food for inmates. As described by former inmates during in-depth interviews, friends and family may bring food items with them during visits, may deliver items without visiting or may in some cases donate cash to an account for use in a facility’s commissary. Alternatively, inmates can participate in informal and semiformal economies through barter and trade with other inmates. Inmates may trade services, or may barter items (both legal and illicit) that they receive from friends and family or make themselves (such as handicrafts and herbal remedies). Though our behavioral-demographic questionnaire contained items asking about trade as a source of income for inmates, reporting was extremely low, and multiple participants commented that these items were likely to be unreliable, because they asked explicitly about outlawed behavior. Nevertheless, each of these avenues for procurement of supplemental food relies to some extent on material assistance gained through social networks.

Social networks have been defined as “the web of social relationships that surround an individual and the characteristics of those ties,” and social integration can be seen as encompassing both the structural aspects such as size and density of an individual’s social networks and the functional aspects such as the frequency, duration and support yielded by the relationships composing a social network [28]. Across populations, social integration has been associated with improved health outcomes, including decreased all-cause mortality [31]. However, social integration can influence individual outcomes through many psychosocial and sociological mechanisms, and the role of material assistance in social network effects remains an area of active research [28], [30].

In some vulnerable populations, comprehensive poverty reduction interventions focusing on social and economic support have improved outcomes of TB treatment and TB contact investigations [19], [21]. However, the health effects of social integration have not been well characterized in confined settings. For incarcerated populations, maintaining social bonds with friends and family outside their facility was associated in one previous study with worse mental health outcomes [29], but more recently isolation from social networks was associated with increased risk of near-fatal suicide attempts [32]. Furthermore, some TB program managers have raised concerns about perverse economic incentives arising from the informal social hierarchy among inmates, which might interfere with TB treatment in former Soviet prisons [39].

In contrast with these concerns, we found that social integration both with friends and family outside the prison facility, and with other inmates, was associated with improved access to supplemental nutrition, which itself was negatively associated with active TB in our sample. The association of higher BMI with visits and deliveries from friends and family further supported this finding. While individuals with self-reported HIV-infection did report less frequent access to supplemental nutrition, neither HIV status nor injection drug use appeared to impact social integration. Duration of incarceration, by comparison, was associated with increased ability to rely on fellow inmates and decreased frequency of visits and deliveries from friends and family. It is important to note that while subjects reported that TB would likely worsen an inmate’s relationships to other inmates, a diagnosis of TB was not directly associated with decreased ability to rely on other inmates for help.

These findings suggest that resources allocated for food provisions should be enhanced in prison facilities in Tajikistan. In addition, programs to augment nutrition among HIV positive and/or socially isolated inmates and comprehensive poverty reduction programs for families of inmates should be investigated as components of TB control policy more broadly. We did not explore the interplay between informal hierarchies and material assistance within inmate social networks in this study. More research is needed to characterize these dynamics and to further elucidate the role of social integration among former Soviet prison inmates in TB epidemiology and health promotion.

### Limitations

The cross-sectional nature of this study challenges the interpretation of our results. Though we have been able to find factors associated with an increased prevalence of active TB in our sample, it is not possible to infer a causal relationship between these factors and the development of active disease. For example, the association of low BMI with active disease in our sample is likely to be due at least in part to the anorexia, which is a cardinal symptom of TB, rather than simply reflecting poor nutritional status. Furthermore, as discussed in detail elsewhere, because of the high mobility of incarcerated populations, prevalence of active disease can vary widely over time and a single point-prevalence is not a stable measure of TB burden in prisons [40]. It is unclear, therefore, what role the risk factors we identified may play in the dynamics of TB epidemiology within prison facilities, or whether our results merely reflect a snapshot of vulnerability to active TB among those most at risk of incarceration.

Our study was also substantially limited by the low proportion of culture-positive cases in our sample. The majority of TB cases identified in this study were diagnosed clinically, on the basis of radiographic findings, symptoms and sputum smear microscopy, and only 27.1% were culture-confirmed. Because quality-assured mycobacterial culture was available only at the National Reference Laboratory for Tuberculosis in the republic’s capital, Dushanbe, sputum samples had to be transported by airplane, and the resulting delays likely limited the accuracy of sputum culture for diagnosis of active disease. However, the possibility of over-diagnosis on the basis of clinical criteria, due either to alternative diagnoses (non-tuberculous mycobacterial disease [NTM], cancer etc.) or to past disease cannot be definitively excluded. No NTM was isolated in culture from any participants, but follow up for the present study did not include data regarding treatment response and any further workup for alternative diagnoses that may have occurred subsequently. Therefore, associations found in our regression analyses should be interpreted in this context, and further research is needed to replicate these findings in settings where more cases can be confirmed with culture.

### Conclusions

As reported in other settings, HIV-infection was associated with increased odds of having active TB in our sample. Policies targeted toward reducing transmission of HIV-infection, especially among injection drug users, may therefore be effective at alleviating the burden of TB in Tajikistan’s prisons. The protective effect of access to supplemental nutrition has not been previously reported in prisons of the former Soviet Union. Identifying inmates at risk for malnutrition and providing comprehensive support may be another avenue for reducing the burden of disease. Further research is needed to further elucidate the role of social networks and social isolation in access to food among inmates.
